# Stakeholder perspectives on proposed policies to improve distribution and retention of doctors in rural areas of Uttar Pradesh, India

**DOI:** 10.1186/s12913-021-06765-x

**Published:** 2021-09-29

**Authors:** Veena Sriram, Shreya Hariyani, Ummekulsoom Lalani, Ravi Teja Buddhiraju, Pooja Pandey, Sara Bennett

**Affiliations:** 1 University of British Columbia, School of Public Policy and Global Affairs and School of Population and Public Health, C. K. Choi Building, 251 – 1855 West Mall B.C, Vancouver, V6T 1Z2 Canada; 2 Johns Hopkins Bloomberg School of Public Health, Ratan Square, Vidhan Sabha Marg, Lucknow, Uttar Pradesh, India; 3 Johns Hopkins Bloomberg School of Public Health, 615 N. Wolfe Street, Baltimore, MD USA; 4 Uttar Pradesh Technical Support Unit, India Health Action Trust, Ratan Square, Vidhan Sabha Marg, Lucknow, Uttar Pradesh India; 5 Indian Administrative Service, Lucknow, Uttar Pradesh India

## Abstract

**Background:**

In India, the distribution and retention of biomedical doctors in public sector facilities in rural areas is an obstacle to improving access to health services. The Government of Uttar Pradesh is developing a comprehensive, ten-year Human Resources for Health (HRH) strategy, which includes policies to address rural distribution and retention of government doctors in Uttar Pradesh (UP). We undertook a stakeholder analysis to understand stakeholder positions on particular policies within the strategy, and to examine how stakeholder power and interests would shape the development and implementation of these proposed policies. This paper focuses on the results of the stakeholder analysis pertaining to rural distribution and retention of doctors in the government sector in UP. Our objectives are to 1) analyze stakeholder power in influencing the adoption of policies; 2) compare and analyze stakeholder positions on specific policies, including their perspectives on the conditions for successful policy adoption and implementation; and 3) explore the challenges with developing and implementing a coordinated, ‘bundled’ approach to strengthening rural distribution and retention of doctors.

**Methods:**

We utilized three forms of data collection for this study – document review, in-depth interviews and focus group discussions. We conducted 17 interviews and three focus group discussions with key stakeholders between September and November 2019.

**Results:**

We found that the adoption of a coordinated policy approach for rural retention and distribution of doctors is negatively impacted by governance challenges and fragmentation within and beyond the health sector. Respondents also noted that the opposition to certain policies by health worker associations created challenges for comprehensive policy development. Finally, respondents believed that even in the event of policy adoption, implementation remained severely hampered by several factors, including weak mechanisms of accountability and perceived corruption at local, district and state level.

**Conclusion:**

Building on the findings of this analysis, we propose several strategies for addressing the challenges in improving access to government doctors in rural areas of UP, including additional policies that address key concerns raised by stakeholders, and improved mechanisms for coordination, accountability and transparency.

**Supplementary Information:**

The online version contains supplementary material available at 10.1186/s12913-021-06765-x.

## Introduction

Ensuring the availability of high-quality, affordable health services for populations living in rural areas is a major policy challenge in many low- and middle-income countries (LMICs). In India, the distribution and retention of doctors to serve in rural areas is an obstacle to improving access to health services. Approximately two-thirds of the Indian population lives in rural areas, while 66% of doctors and 64% of health workers more broadly live in urban areas [1]. The vast majority of these doctors, 80.4%, work in the private health sector [1]. Recruiting and retaining doctors to work in the government sector, specifically to boost the availability of affordable health services for poor and vulnerable populations living in rural areas, is a longstanding policy issue.

In this paper, we focus on the development of human resources for health (HRH) policies to address rural distribution and retention of doctors in the public sector in the northern Indian state of Uttar Pradesh (UP). Using a prospective stakeholder analysis, our objectives are to 1) analyze stakeholder power in influencing the adoption of policies; 2) compare and analyze stakeholder positions on specific policies, including their perspectives on the conditions for successful policy adoption and implementation; and 3) explore the challenges with developing and implementing a coordinated, ‘bundled’ approach to strengthening rural distribution and retention of doctors. We focus specifically on policies to attract qualified doctors trained in allopathic medicine to work in rural areas, recognizing that a significant proportion of doctors in rural areas are either unqualified biomedical providers or trained in traditional forms of medicine.

The situation in UP reflects many of the challenges with rural distribution and retention of doctors in India. The state has approximately 4.9 doctors per 10,000 population, below than the national average of 5.9–6.1 [2]. Further, major maldistributions exist between private and public sectors. For example, 80% of specialist posts in the government sector are vacant [3]. Within the public sector itself, there is a significant variation in the distribution of doctors across the state. For example, Siddharthnagar, a rural district, has 32 doctors per million population while Lucknow, the largest city, has 188 doctors per million population [4]. From a governance standpoint, the responsibility for HRH in UP, like many other Indian states, is heterogenous. HRH policy is spread across at least four different state level directorates (Medical Education, Medical Health, Family Welfare, and traditional medicine) as well as the central government through the National Health Mission (NHM), which directly employs approximately 40.5% of health workers and 26% of medical officers in UP, and provides additional funds for medication, equipment, supplies, etc. [4]. Many of these challenges, particularly the maldistribution of skilled health workers between rural and urban areas, and high vacancies in rural areas, mirror the problems faced in other LMICs [5, 6].

**Fig. 1:**
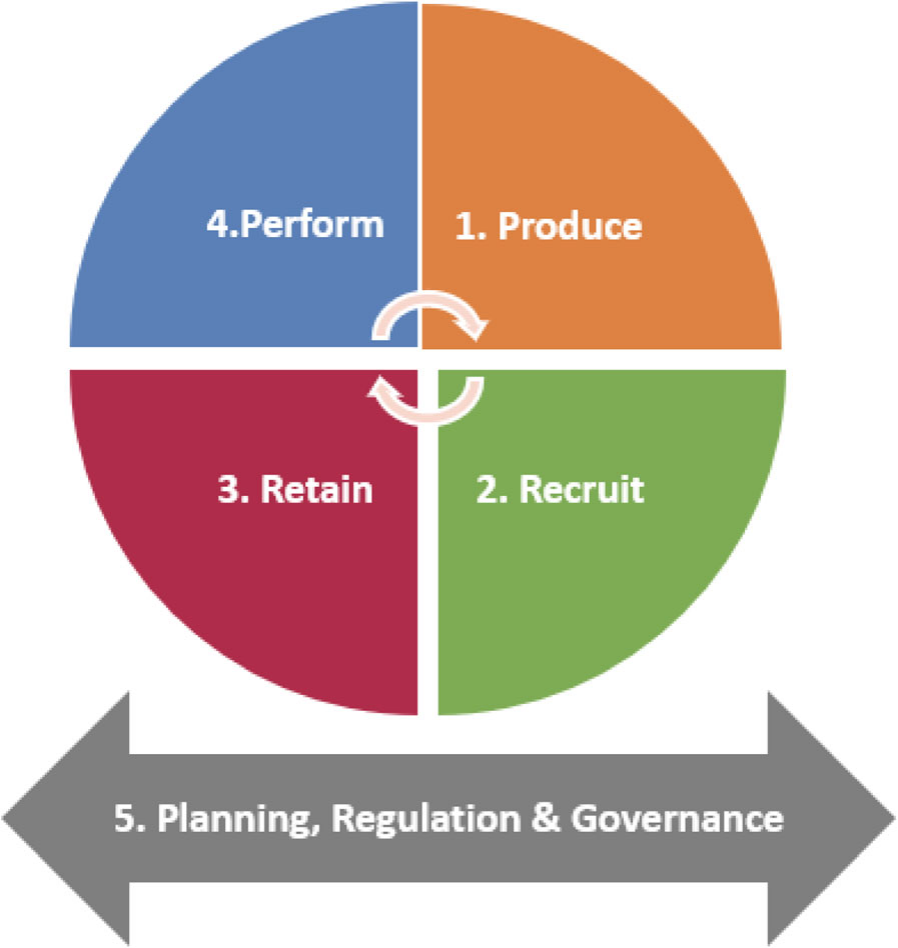
Life cycle approach to human resources for health policy development

The Government of Uttar Pradesh (GoUP), in coordination with the UP Technical Support Unit (UPTSU) and the Johns Hopkins Bloomberg School of Public Health, is developing a comprehensive HRH strategy to guide policy development in the state over a ten-year period, which includes policies to address the distribution and retention of doctors in underserved and rural areas of the state. This HRH strategy builds on existing efforts taken by the state to address rural distribution and retention of doctors including increasing the number of medical colleges in semi-urban or rural districts, improving transparency in human resource processes, non-financial incentives and compulsory rural service bonds [7]. The strategy is built around the ‘lifecycle’ approach of HRH and focuses on five main domains – production, recruitment, retention, performance and cross-cutting governance (Fig. 1). This approach is informed by multiple HRH frameworks that examine health workforce performance and processes across the HRH lifecycle [8].

A key objective of the strategy is to ‘promote distribution of health workers to rural and underserved areas’ in the state. Achieving this objective requires a ‘bundle’ of interventions that cut across these different domains [9, 10]. Table 1 outlines an initial list of proposed policies to reach this objective around rural distribution and retention, alongside explanations for why these policies were selected. All initial policies were identified by GoUP, UPTSU and Johns Hopkins Bloomberg School of Public Health based on existing priorities and policy initiatives for GoUP, an examination of state-of-the-art literature, and discussions with key informants.^1^ Certain ongoing policies, such as the allocation of additional points for postgraduate entrance examinations, were not identified during exploratory work as warranting further improvements at the time of the study.

We conducted a stakeholder analysis to gather perspectives on the draft strategy from key stakeholders, with the goal of guiding more effective policy development and implementation. Stakeholder analyses are a common tool in policy development [11–13], but less frequently utilized in examining multi-pronged policy strategies, including in HRH policy. The few stakeholder analyses of HRH policymaking provide key insights, including the impact of power dynamics, defined here as the relative capacity to influence policy decisions in a particular context [14–18]. Examples of power dynamics in this literature include the critical role of health worker associations in shaping policy processes, tensions between bureaucrats and health workers in policy prioritization, and interference from politicians at multiple levels of decision-making [14, 15, 17]. The literature also suggests that addressing rural retention of health workers through ‘bundled’ strategies [9, 10, 19], needs careful attention to governance processes, as well as the perspectives of health worker associations [14]. Given the range and complexity of policies collectively needed to improve rural retention of doctors and other health workers, more stakeholder analyses are needed to explore a variety of policy options from multiple perspectives, with the goal of facilitating more effective policy development.

In this paper, we share findings from our stakeholder analysis of HRH policy in UP, focusing specifically on the rural distribution and retention of biomedical doctors in the public sector of UP. Our paper is structured as follows – first, we describe stakeholder power in influencing the adoption and implementation of policies; second, we compare and analyze stakeholder positions on specific policies and their views on the conditions needed for successful policy adoption/implementation; and finally, we present cross-cutting challenges to developing a coordinated, ‘bundled’ approach to strengthening rural distribution and retention of doctors in this context.

**Table 1:** Proposed policies to improve rural distribution and retention of doctors in Uttar Pradesh

Policy	Rationale
1. Increase opportunities for students from rural backgrounds to enter medical college in the public sector, through providing state sponsored training for the NEET for students from rural areas. (Production)	• Since 2016, medical college aspirants in India must take the National Eligibility cum Entrance Test (NEET) and score within a specified percentile in order to secure admission. • However, reports have found the exam to perpetuate inequities in medical college admissions, such as favoring students from wealthier, urban backgrounds [22]. • The success of these students is also driven by their ability to afford private ‘coaching’ for the examination through a pervasive, profit-driven coaching industry [55]. • Research suggests that medical students from rural areas are more likely to return to serve in rural areas [23], thereby raising questions about the challenges of recruiting rural students in the current system of NEET coaching. • Government sponsored NEET coaching has been announced by the Delhi Government and the National Training Authority, and by the Government of Tamil Nadu [24, 25]. The policy proposes that GoUP similarly create opportunities for students from rural backgrounds to enter medical professions.
2. Modify and enforce a compulsory rural posting policy requiring that all newly hired doctors spend a minimum of 3 years in an underserved community (priority districts/underserved rural facilities) so as to increase the availability of health staff in rural areas, after which they have the option to transfer to another posting. (Recruitment)	• Compulsory service programs have been used to deploy and retain health workforce in rural areas in many countries and have demonstrated varied success [26–28]. • GoUP required newly hired doctors to be posted to a Primary Health Center at the discretion of the District’s Chief Medical Officer (CMO). However, key informants indicated that these policies were poorly enforced and subject to informal influence. These factors provided new medical officers the ability to circumvent requirements and be posted to higher level facilities. • To address these influences, the proposed modification of this policy builds on recent changes which centralize the posting of new recruits by prioritizing rural areas postings with a minimum duration of 3 years. Upon fulfilling these requirements, recruits may transfer to another posting.
3. Allow home district posting for clinical cadres (Retention)	• Home district posting is restricted for a large number of government cadres across Indian states including doctors and staff nurses in UP as a means to reduce conflict of interest with the quasi-judicial and financial powers associated with this category of government posts. • There is growing consensus that this should not be applied to clinical cadres like doctors and nurses who have limited responsibilities that put them in a position of a conflict of interest, instead, clinical cadres are also likely to be more accountable when serving their own communities. • Key informants noted that home district posting could further contribute to greater availability of health workers in rural posts given that such students are more likely to go back and serve in their communities [23]. This will also address gaps in recruiting more students from rural backgrounds for medical training. • Furthermore, several Indian states, including Gujarat, have also relaxed home district posting to address rural shortages [29].
4. Living conditions – Improve staff housing infrastructure and security in rural facilities (Retention)	• In discussions with key informants, appropriate living conditions, including the ability to live with family, emerged as an important motivation for health workers in UP in choosing a posting. • These factors have also been identified as key in other states in India [20]. For example, graduating medical students in the state of Odishaviewed good housing and adequate facilities as key to attracting more students toward rural service [30]. • While GoUP currently has policies and allocation of funds for infrastructure improvements, including housing for health workers, key informants noted that implementation remains a challenge. • Group housing for health workers to enable them to live with their families and have access to basic amenities and security while working in far flung areas has been a policy option that states like West Bengal, Uttarakhand and Chhattisgarh have used [28]. GoUP may consider a similar policy to improve living conditions for health workers in rural areas.
5. Working conditions - Increase coordination across health departments and agencies to ensure that health workers have appropriate inputs and supports to do their job including the availability of functioning equipment, electricity, drugs and other supplies. (Retention)	• The conditions in which doctors work – such as supplies, drug and equipment availability, electricity, water and other utilities – are major issues for doctors to contend with upon entering government service, particularly in rural areas [28]. • Studies across multiple Indian states have found working conditions to be an important factor in retaining staff in rural areas [30, 31]. • The UP Directorate of Medical and Health, UP National Health Mission, and the UP Medical Supplies Corporation have policies and resources to ensure adequate working conditions, however, key informants suggested coordination across different agencies and levels was essential yet often neglected.
6. Permit private practice for government doctors and develop a policy that regulates hours and conditions under which private practice can occur and remove the non-private practice allowance. (Retention)	• Dual practice is a widespread phenomenon in LMICs, and is also used as a retention strategy to retain highly-skilled physicians in the public sector [32]. • Negative impacts associated with dual practice include absenteeism or fatigue among health workers, and diversion of patients to private clinics [33]. • In India, dual practice is widely believed to be common, and high rates of absenteeism have also been inferred as indicative of dual practice [34]. • In UP, private practice is not permitted and government doctors receive an NPA (non-practicing allowance), which currently forms 20% of a medical officer’s basic pay, to compensate doctors. However, it is perceived by some key informants that this rule is widely flouted and a majority of government doctors pursue some form of private practice while receiving NPA. • Several other Indian states have a similar ban on private practice along with an NPA [35]. However, there are exceptions. In the state of Kerala, public doctors can see patients on a ‘private fee-for-service’ basis outside their government work time [34, 35].
7. Support the shifting of tasks from MBBS to AYUSH doctors in rural settings: i. Assess the clinical competence of AYUSH doctors in the provision of services previously provided by MBBS ii. Provide additional on-the-job and short-term training to AYUSH doctors iii. Ensure supportive supervision to AYUSH doctors iv. Explore further task shifting options for Specialists to MBBS and MBBS to nurses (Planning, regulation and governance)	• Task-shifting has been used in many countries and in several Indian states to address serious workforce shortages of health workers [36–38]. • In some Indian states, AYUSH providers are co-located with allopathic doctors in primary health centers, and in recent years, have been permitted to prescribe a limited range of allopathic medications [36, 37]. • In UP, there has been some movement to broaden task shifting of biomedical services to AYUSH providers in rural PHCs. Qualified nurses have more recently been engaged as Community Health Officers at Health and Wellness Centres. • Key informants suggested that further training and monitoring of task shifting to AYUSH was required in the UP context.

## Methods

We conducted a prospective stakeholder analysis using qualitative methods. Qualitative methods are commonly utilized in stakeholder analyses in order to understand individual and organizational policy positions, and to examine stakeholder power, interests and relationships [11, 20]. The research team consisted of researchers from the Johns Hopkins Bloomberg School of Public Health (based in Lucknow and in the U.S.) and UPTSU, with support from GoUP officials.

We utilized three forms of data collection for this study – document review, in-depth interviews and focus group discussions.

1. **Document review:** We examined key documents pertaining to HRH policy in UP, including government policies, service rules, directives, reports and news articles. A selective review of national level HRH policies was undertaken along with exploring the experiences of other states with particular reforms.

2) **Interviews and focus groups:** We conducted semi-structured interviews and focus groups with key stakeholders engaged with human resources for health policy in UP. We implemented the following steps –

### Stakeholder mapping and sampling

The research team drew upon a priori knowledge to iteratively develop a mapping of stakeholders with interests in the development and implementation of the various policies in the strategy. In the mapping, we prioritized stakeholders to groups of high, medium and low relevance to policies in the strategy. We conceptualized relevance as stakeholders who play a definitive role in the policy process, for example, by influencing the adoption of a policy in initiating, supporting or blocking a policy, or influencing its implementation. We then held detailed discussions regarding each of the 57 listed stakeholders and utilized purposive sampling [21] to select stakeholders to contact for interviews or focus groups based on four parameters: 1) relevance to the HRH strategy; 2) diversity of stakeholder categories (government, professional associations, civil society, etc.); 3) ability to arrange and organize interviews/focus groups through networks, and 4) geographic proximity to Lucknow (the location of the majority of the team).

### Interview and focus group guide development

The interview and focus group guides were developed based on guidance from Schmeer et al. (1999) [56], and focused on capturing four main points of data as they pertained to policies listed in Table 1, stakeholder positions, interests, power, relationships, and views on conditions for successful policy adoption/implementation, as well as overall perceptions of HRH challenges in UP (see supplemental data for interview and focus group guides). The findings reported in this paper represent a portion of the policies in the HRH strategy; interviews conducted with respondents covered policies beyond rural retention and distribution. Given the number of policies, we shared the full listing of policies with stakeholders and invited them to comment on those policies most relevant to their positions and interests (as determined by the respondents and the research team).

### Data collection

Following the sampling process, we pursued interviews and focus groups with 33 stakeholders. Seventeen interviews and three focus groups were conducted by SH, VS and/or RB (Table 2 provides the categorization of respondents). We could not get appointments with three stakeholders, one stakeholder declined, and we had informal discussions with nine stakeholders (the data from which are not formally reflected in this paper but informed our background thinking). Data collection took place between September and November 2019, in Lucknow, New Delhi and one rural district in Uttar Pradesh (name withheld for confidentiality).

**Table 2:** Respondents interviewed for stakeholder analysis

	Interviews	Focus groups
1. Department of Medical Education (current and former officials)	IDI3, IDI4	
2. Department of Medical Health and Family Welfare (current and former officials)	IDI9, IDI12	
2a. Frontline medical officers		FGD1 (Frontline medical officers, n = 6)
2b. Frontline administrators	IDI16	FGD3 (District-level administrators, n = 3)
3. Training institutes	IDI7	
4. Health worker associations (doctors, nurses, traditional medicine doctors, etc.)	IDI6 (Doctors’ association), IDI8 (Doctors’ association), IDI13 (Traditional medicine providers’ association) IDI14 (Rural providers’ association)	FGD2 (Members of state nurses association, n = 13)
5. Private sector hospital association	IDI5	
6. Civil society organizations	IDI11	
7. Regulatory agencies	IDI2	
8. National level agencies	IDI10, IDI17	
9. Health policy and systems experts	IDI1, IDI15	
Total	17	3

* Certain respondents held multiple positions within the government, but are categorized under their primary designation

Interviews and focus groups were conducted in private rooms to ensure confidentiality. Participants for focus groups were recruited based on existing networks of the research team and snowball sampling. Front line medical officers (*n* = 6) from a diverse range of districts within UP were recruited during a training program at the state-level training center. Frontline nurses were recruited from the main nurses’ association in the state (*n* = 13). District-level administrators (*n* = 3) in rural district were recruited from the networks of research team members. All but one interview/focus group was audio-recorded, with the permission of the respondent(s). Interviews were conducted in English or Hindi (or both), and lasted between 15 min to three hours. Focus groups were conducted in Hindi, and took approximately one to two hours.

### Analysis

Audio recordings were translated and/or transcribed into English, cleaned and checked. VS, SH, UL and SB developed a codebook through several rounds of line-by-line coding. The codebook and coding process was finalized through a round of coding in which two analysts reviewed the same transcripts, cross-checked their coding and discussed differences in approach. Finally, the transcripts were divided among VS, SH and UL for the final round of coding. Codes were added, deleted or modified throughout the coding process, and regular team meetings were held to discuss the coding process, the codebook and early ideas for theme development. After all coding was complete, VS, SH and UL began extracting data specific to rural retention and distribution and categorized them into three main areas – context, stakeholder power and interests, and stakeholder positions on policies. VS, SH, UL and SB developed themes pertaining to barriers and facilitators through repeated analysis of the coded data.

This research was considered non-human subjects research by the Johns Hopkins Institutional Review Board (Baltimore, MD; IRB No: 00009035) and by Sigma Research and Consulting (New Delhi, India; IRB No.: 10040/IRB/19–20).

## Results

The results are structured as follows – first, we outline the stakeholders interviewed for this project, their interests as they pertain to the issue of rural distribution and retention, and their power relative to other stakeholders; second, we describe the position of these stakeholders regarding the policies and the reasons behind their positions; and third, we explore issues pertaining to developing a coordinated strategy for rural distribution and retention.

### Stakeholder interests and power

Table 3 describes the interests, power and policy influence of the stakeholders interviewed for this study. The UP Department of Medical Health and Family Welfare^2^ (comprised of various Directorates and centrally-funded programs such as the National Health Mission) was considered most powerful by respondents in terms of developing and implementing policy. However, respondents also noted that groups such as doctors’ associations had considerable ability to oppose policy and impede their adoption.

**Table 3:** Stakeholder interests and power of respondents interviewed

	Interests (Extent to which a stakeholder is impacted or affected by a change in policy) [22]	Power (Potential capacity to influence policy decisions in this context) [23]
**1. Department of Medical Education, Uttar Pradesh**	Increasing numbers of available health personnel through expansion of undergraduate and postgraduate education, increasing exposure to rural contexts, establishing/administering medical colleges, complying with regulations	Moderate influence in expanding seats and improving quality, due to involvement of national-level authorities, such as National Medical Commission; high influence over setting health worker renumeration, ensuring coordination with Medical and Health Department.
**2. Department of Medical Health and Family Welfare, Uttar Pradesh (i.e., Directorate of Medical and Health Services, National Health Mission and other central government programs)** **2a. Frontline medical officers (i.e., government medical officers working in primary and community health centers)** **2b. District-level administration (i.e, Chief Medical Officers, Additional Chief Medical Officers, NHM staff)**	Increasing numbers of available health personnel in rural areas, coordinating stakeholders at the state level and with the central government Ability to provide services effectively; ensuring proper renumeration and working/living conditions in rural areas; career pathways Managing health services within the district, ensuring adequate staffing, managing performance, liaising with higher levels within Medical and Health	High ability to initiate and implement policy pertaining to the public sector health system, including renumeration, performance management and transfers Ability to organize through public doctors’ medical associations – however, views and priorities of rank-and-file members might not be reflected in positions taken by association leadership High ability to exercise discretion in implementing policy decisions at the district level, but lesser ability to influence the policy adoption
**3. Regulatory agencies (i.e., Uttar Pradesh Medical Council, U.P. State Medical Faculty)**	Managing quality of medical, nursing and allied health worker education, liaising with national level regulators in the case of nursing and allied health education	Moderate level of power in nursing and allied health worker education; considerable ability to improve quality of nursing and allied health worker education
**4. Private sector hospital associations**	Responsible for representing interests of private sector nursing homes and hospitals including those facilities in rural areas; advocating for inclusion of unregulated health workforce	Moderate levels of power relative to decision-makers, and consists of sizeable membership
**5. Doctors’ associations (i.e., government doctors’ association, combined public and private doctors’ associations)**	In the private sector, advocating for policies in support of doctors’ interests and opposing regulation on their practices; in the public sector, ensuring appropriate living and working conditions, advocating for functioning HR processes for members such as promotions, benefits, etc. (public and/or private sector depending on the association); in both sectors, maintaining relative power of medical profession vis-à-vis traditional providers or non-qualified doctors	Moderate levels of power relative to decision makers, but higher level of power compared with other health professions and occupations; ability to mobilize and advocate for policy positions through mechanisms such as strikes, etc.
**6. Nurses’ associations**	Ensuring acceptable levels of reimbursement, and working and living conditions for nurses; advocating for functioning HR processes for members such as promotions, benefits, etc. (primarily in the public sector)	Moderate levels of power relative to decision makers, but lesser ability to influence policy compared with doctors; sizeable membership and significant ability to mobilize and organize
**7. AYUSH doctors’ associations**	Advocating for AYUSH health professionals in the public and private sector, ensuring AYUSH providers receive adequate training and support in the context of task shifting and other policies, advocating for other HR benefits.	Lower levels of power relative to biomedical professional associations and decision-makers
**8. Rural medical practitioners association**	Advocating for health professionals serving in rural areas (from different backgrounds - allopathic, traditional, etc.) and uniquely representing those private sector providers serving in rural areas with limited options for health care	Lower levels of power relative to biomedical professional associations and decision-makers
**9. National-level health agencies (i.e., national-level government health agencies and institutions, quasi-government think tanks, etc.)**	Supporting distribution of health professionals in rural areas through policy development, financial support, support for implementation, and monitoring and evaluation	High levels of power in terms of policy prioritization, but cannot directly manage state-level policies for HRH; certain national level groups can control NHM workforce distribution within the state
**10. Civil society organizations**	Ensuring access to health services in rural areas, supporting human rights-based approaches and community engagement in health service delivery/oversight	Moderate levels of power relative to decision makers due to direct access to communities, but infrequently engaged by policymakers on HRH policy

### Policies and stakeholder positions

Table 4 outlines positions for each of the policies (supportive, supportive with reservations or opposed) and perceived barriers and facilitators for adopting the policies.

**Table 4:** Stakeholder positions on policies and implementation considerations

Supportive	Reservations about the policy	Opposed
**Policy 1:** Increase opportunities for students from rural backgrounds to enter medical college in the public sector, through providing state sponsored training for the NEET for students from rural areas.		
Department of Medical Education, regulators, training institutions, doctors’ association, AYUSH association, medical officers, district-level administration	Medical officers, Private sector hospital association, (personal opinion, did not represent association)	
**Policy 2:** Develop and enforce a compulsory rural posting policy requiring that all newly hired doctors spend a minimum of 3 years in an underserved community (priority districts/under-served rural facilities)		
Private sector hospital association, rural medical practitioners association, national level agencies, civil society	Doctors’ associations (leadership was personally supportive)	Frontline medical officers, district-level administrator, regulators
**Policy 3:** Allow home district posting for clinical cadres (RETAIN)		
Doctor’s associations, nurses’ association, medical officers, national-level stakeholders		District-level administration
**Policy 4:** Living conditions – Improve staff housing infrastructure and security in rural facilities. (RETAIN)		
All stakeholders		
**Policy 5:** Working conditions - Increase coordination across health departments and agencies to ensure that health workers have appropriate inputs and supports to do their job including the availability of functioning equipment, electricity, drugs and other supplies. (RETAIN)		
Most stakeholders	Health systems expert	
**Policy 6:** Permit private practice for government doctors and develop a policy that regulates hours and conditions under which private practice can occur and remove the non-private practice allowance. (RETAIN)		
Medical officers	District-level administration, doctors association	National-level stakeholders; civil society
**Policy 7(a)** Support the shifting of tasks from MBBS to AYUSH doctors in rural settings through: i. Assess the clinical competence of AYUSH doctors in the provision of services previously provided by MBBS ii. Additional on-the-job and short-term training to AYUSH doctors Supportive supervision to AYUSH doctors		
Directorate of Medical Education, AYUSH association, rural medical practitioners association, national-level heath agencies	Medical officers	Doctors’ association, district-level administration
**Policy 7(b)** Explore further task shifting options from MBBS to nurses (community health officers)		
Nurses associations, national level health agencies, frontline health workers, Department of Medical Education, civil society organization	Doctors’ association

Policy 1: Increase opportunities for students from rural backgrounds to enter medical college in the public sector, through providing state sponsored training for the NEET for students from rural areas (PRODUCE).

Respondents across stakeholder categories were largely supportive of the policy measure, with some recognizing inequities in the current privately-driven system of preparatory ‘coaching’ for NEET. However, several respondents who were supportive also expressed hesitancy and uncertainty about implementation and overall success. Two factors appear to be driving this hesitancy. The first was a sense from doctors at the frontlines, such as medical officers and medical college faculty, that there would be practical difficulties in organizing such a program, and that existing challenges would remain for rural students upon entering medical college. For example, respondents in a focus group of medical officers noted that state-sponsored programs would not be of high-quality and that students should instead be given discounts to utilize private coaching centers.

*“If the coaching centers are started by government sector then I don’t think they would be able to deliver high quality students....”* Medical officer, FGD01 Frontline medical officers.

*“I think that if the student is from the rural background then they can get a discount in the fees which they have to pay at any coaching center where they want to study....For example if the fees is Rs 40000 in the private coaching they should just have to pay Rs 10000 for the same coaching....”* Medical officer, FGD01, Frontline medical officers.

Some respondents also cautioned that even if the NEET training was successful, rural students face numerous educational and social barriers upon entering medical college, such as the widespread use of English-language textbooks, a challenge given limited English training in rural schools in UP, and broadly, poorer quality basic education in rural areas across the state.

Second, some respondents also questioned the underlying premise that individuals from rural areas would want to go back to those areas upon graduating (IDI7, IDI1, IDI16).

“*… if a person has spent his whole life in poverty why will he go back to that? He doesn’t go back. Your purpose is to provide the opportunity to people who belong to rural background so that they can again go back in that society but they will never go back*.” IDI1, Health systems expert.

Finally, a few stakeholders, most notably frontline health workers, noted that there were existing policies to increase the number of rural students in medical colleges, such as reservations (IDI2, IDI7, FGD1).

Policy 2: Develop and enforce a compulsory rural posting policy requiring that all newly hired doctors spend a minimum of three years in an underserved community (priority districts/under-served rural facilities) so as to increase the availability of health staff in rural areas, after which they have the option to transfer to another posting (RECRUIT).

Respondents were strongly divided on this policy. Medical officers largely opposed it, highlighting difficult living and working conditions for staff in many rural facilities.

*“Suppose your salary is Rs 60000 and you have to spend Rs 20000 on traveling to the rural areas and in case you face any problem then you have to pay money to babus (local administrators) to sort it. So ultimately the money which is left is just Rs 40000 and on top of that there is no infrastructure and if you have to go to the field then you have to travel on your own.”* FGD1 (Frontline medical officers).

These views were shared by other respondents working at the district or sub-district level. These respondents noted that compulsory postings have not worked in this context so far, and that the high levels of vacancies and absenteeism is indicative of the problems with this approach. One regulator noted that there was no ‘political will’ to take this forward, and that policymakers should instead focus on posting practitioners of traditional forms of medicine -.

Ayurveda, Yoga & Naturopathy, Unani, Siddha, Sowa-Rigpa and Homoeopathy (AYUSH) in rural facilities.

*“There are 2 things. One is by force and the other by option. Both are being used. Well, both options are okay. But in the long run if the people opt in themselves only then will it be successful. In the short run it can be successful by force but not in the long run. People are going to opt for it when the overall package will seem attractive to them”* IDI16, District-level admin.

In contrast, however, respondents who served in policy and decision-maker roles supported the policy and suggested that it had to be broadened in its scope. For example, a health systems expert added that specialists also needed to perform some form of mandatory rural service.

Other respondents noted that while the policy was needed, successful implementation would be a challenge, as seen by the current levels of absenteeism and absconding.

Doctors’ associations had mixed responses. The respondents interviewed *personally* felt that the policy would be appropriate but felt that the associations could contest it – for reasons that include inadequate working and living conditions. One respondent talked about how personal views could become mixed with association viewpoints.

*“Even sometimes [doctors’ association] also oppose because I am a parent I cannot say directly and so I will say indirectly through [doctors’ association] saying this policy should not be implemented. But this policy is good if we want to give services to rural people, then this policy is good.”* IDI8, Doctors Association.

Policy 3: Allow home district posting for clinical cadres (RETAIN).

Respondents, particularly doctors in the public sector, seemed broadly supportive of the policy. Doctors noted that home district posting would boost recruitment and retention by allowing medical officers to stay with their families, secure educational opportunities for their children, and attend to their parents. Finally, some respondents noted that individuals in their home districts would be less likely to abuse their position and take or accept bribes.

*“... if you send me to Agra or Azamgarh, then no one knows me there, so, I can do anything there, I will take 100 rupees for an injection, I will take so much money and scam a lot of people. but if I live in my own district, then people will say that, she is that person’s daughter and she brought shame to her family, she takes 100 rupees for an injection. So, I would not take money because of that fear and I will not talk rudely with anyone.”* FGD2 (Nurses’ association).

However, district-level administrators noted the negative implications of allowing home district posting, in terms of limited accountability from their community members, abuse of their office through private practice, and potential lack of objectivity in medico-legal cases.

*“The reason is if you allow home district posting then the person becomes audacious. He will not work-- he would put local pressure and prefer to stay at home mostly than work.”* FGD 3 (District-level administrators).

Policy 4: Living conditions – Improve staff housing infrastructure and security in rural facilities. (RETAIN).

Respondents widely agreed that improving living conditions for health workers, particularly in terms of housing and security, was essential to improving rural distribution and retention.

“*There is no fan or electricity and even if is there and if it goes off nobody is there to repair it and if you need it very much you can call your own electrician and pay your own money and get it repaired.”* Medical officer 4*,* FGD1 (Frontline medical officers).

Frontline health workers also noted negative mental or physical health impacts due to unsatisfactory or inadequate living arrangements and security provisions. For example, some female staff discussed uncertainties around personal security and safe transportation. Solitary living arrangements were also said to facilitate substance abuse, such as alcohol abuse.

Another challenge stated by stakeholders with direct experience was the diversion of funds set aside for improvements to living arrangements, and inadequate inspections and maintenance of housing infrastructure. The following exchange emerged from the focus group with frontline medical officers:

“R 4: *The budget which comes should be properly distributed and look into it like the budget is wasted at certain places and not utilized properly.*

R 1: *Actually, whatever infrastructure have been developed are 20 years old and no new infrastructure have been developed.*

R 2: *And the quality of infrastructure is poor.*

R 1: *And whatever has been developed is not maintained properly.*

R 2: *There is a lot of corruption in it*.”

Doctors and district-level administrators noted that building a residential colony or township for health workers and other district-level workers from other departments, an approach taken for central government employees and army personnel, would address several challenges. Such colonies or townships could also have regular transportation for health workers.

Policy 5: Working conditions - Increase coordination across health departments and agencies to ensure that health workers have appropriate inputs and supports to do their job including the availability of functioning equipment, electricity, drugs and other supplies. (RETAIN).

Respondents noted that while the Directorate of Medical Health and Family Welfare, with support from NHM, had necessary policy provisions and resources to ensure adequate working conditions, in practice these are not always met. Doctors interviewed noted that addressing gaps in working conditions was also a question of effective management by Chief Medical Officers and other administrators.

*“I was a superintendent of two CHCs for 3-4 years and there was not a single non-functioning equipment and there was not a single case where a lifesaving drug was required and it was not there. Because we are already getting enough funds from NHM and all …*. *So I never faced any funds crunches so basically there is willingness and there are some hospitals which are being maintained very nicely with the same funds. So it depends on whether the CMO is asking.”* IDI6 (Doctors’ association).

In addition, a respondent representing civil society noted the potential role of the Rogi Kalyan Samitees (RKS) or patient welfare committees formed at hospitals/ CHCs and FRUs and their role in ensuring adequate working conditions at the facility.

One challenge raised by an expert was the fact that many of these issues were outside the scope of an HR policy.

*“What can HR policy do for drug availability? I do not think so because both are two different domains...”* IDI1 (Health systems expert).

Policy 6: Permit private practice for government doctors and develop a policy that regulates hours and conditions under which private practice can occur and remove the non-private practice allowance (NPA) (RETAIN).

Respondents widely acknowledged the ineffectiveness of the NPA in preventing private practice, and doctors were largely supportive of the policy measure. Yet, the policy also triggered reactions regarding implementation complexities. Concerns were centred around the removal of the NPA itself, and the regulatory challenges associated with private practice and its potential negative impact on public-sector service delivery.

Government doctors’ association and frontline medical officers, likely operating from a belief in the need to protect the additional income earned through the NPA, believed it was unacceptable to completely remove the NPA from current government doctors and suggested that the policy be made voluntary. Further, the opposition of the government doctors’ association was viewed as sufficient to stall any forced removal of the NPA.

Around the second theme of the ability of government to regulate private practice, a respondent from a national-level agency raised a range of concerns –.

*“… so if I am a private practitioner, I am going to only think about my practice so I won’t come on time, I would not look at the patients that seriously, I perhaps would not even treat them and say that you know I have a clinic in the neighbourhood please come and see, I have seen people who are practicing from their own quarters just adjacent to their hospitals and unless there is an inspection they just don’t go to the hospital …*” IDI10 (National-level health agency).

This respondent instead thought that it would be better to remove the NPA but continue to maintain a ban on private practice while significantly raising government salaries as a retention strategy, as a way of closing the large gap between public and private sector salaries.

Another suggestion offered by district-level administrative officers was to allow private practice but provide performance targets to doctors for their work in public facilities, beyond which they could be free to pursue private-sector work. However, a senior district-level administrator, while agreeing with the policy option on principle, said GoUP was currently poorly capacitated to take on this regulatory function, and thought it better maintain the ban on private practice. Similar sentiments were echoed by the civil society respondent, who further added that even community-based monitoring to regulate private practice would have limited effectiveness in a context like UP, where the power imbalances between community members and doctors would hamper reporting adverse events.

Policy 7(a): Support the shifting of tasks from medical officers to AYUSH doctors in rural settings through:

i. Assess the clinical competence of AYUSH doctors in the provision of services previously provided by MBBS
ii. Additional on-the-job and short-term training to AYUSH doctors
iii. Supportive supervision to AYUSH doctors

(Planning, Regulation and Governance)

Doctors’ associations, district administrators and frontline workers strongly opposed task shifting from medical officers to AYUSH in primary health centers. Government officers and doctors in particular questioned the competence of the cadre, reflecting longstanding distrust of traditional Indian systems of medicine by practitioners of allopathic medicine in India [39]. They also noted that there will be strong opposition from doctors’ associations due to the perception that such policies promote unsafe forms of medicine or ‘quackery’, and also a sense of ‘possessiveness’ about the domain of allopathic medicine.

*“everybody is against it. .. Their argument is this is my domain and nobody should enter it. It is possessiveness, unnecessary possessiveness.” IDI08 (Doctors’ association).*

*“Ayurveda has a whole different system. They have different concepts. Homoeopathy has an altogether different system and different concepts. Straight from the name of diseases to how they occur, everything is different. So, you cannot merely tell him the names of 4 medicines and give him a bridge course. This should not be done.” IDI16 (District-level administration).*

Doctors also mentioned that AYUSH task shifting did not address the root causes of doctor shortages, such as subpar living conditions, safety concerns and corruption.

*“You are not able to attract doctors you are not thinking how to attract doctors you are thinking how to replace them with nonprofessionals this entire logic is fraud. You are saying that we are over burdened with this clinical work and managerial work, so unburden us recruit more, this does not mean that you will start giving our functions to anybody else.” IDI06 (Doctors’ association).*

Respondents supportive of AYUSH task shifting also included the AYUSH doctors’ association and rural medical practitioners. These respondents mentioned the frontline reality of AYUSH providers providing allopathic or integrated care due to the lack of doctors, and noted the limitations faced by this cadre, including the power asymmetry between allopathic and AYUSH providers and the need for better training.

Policy 7(b) Explore further task shifting options from MBBS to nurses (community health officers).

All respondents who discussed this policy option were supportive, but some noted that for it to be successful, proper education and training mechanisms would need to be put in place. Respondents also mentioned the often-poor quality and training of private nursing colleges and additional support systems would be required to address these competencies gaps.

*“What is happening - our nursing colleges in last 10 years were very poor and lot of private nursing colleges have come up who are not training at all, they are just giving the degree. So we do not know which nurses or which type, where they have been trained.” IDI07 (Medical college leadership).*

The nursing association was supportive of this policy under the condition that proper allowances were provided, and that sufficient guidelines and policies were developed for nurses in these positions. Respondents also mentioned absence of policies that protect nurses who are already filling in the gaps during doctor shortages, further highlighting existing structural and power dynamics between the nursing and medical doctors. Some limitations included existing restrictions to prescribe medications and continued opposition from the medical association.

*“Doctor leaves the office at 2 o clock, but we continue to do the dialysis until 7 o clock, there is no doctor there, until DC is not filled, doctors don’t come there, that is the condition of [facility name] and forget about other places … these kids have the full power to give any emergency drug, they have joined us 7 months ago, they have the full power to give injections … they can give everything, they decide whether you have to give oxygen to him or not, you have to use the drip or not.” FGD02 (Nurses’ association).*

National level respondents who were supportive of this policy measure mentioned examples of past successes of MBBS to nurse task shifting and highlighted examples from other countries. Reiterating similar concerns mentioned by nurses’ associations, national level respondents mentioned legal barriers and existing acts which prohibit nurses from prescribing medications.

*“… one option is instead of interfering with each and every legacy Act just have one new Act and new set of rules … So, then that is a much simpler solution. Rather than trying to amend each and every act, because we have so many different Acts which kind of affect this issue.” ID17 (National-level health agency).*

### Cross-cutting challenges in developing and implementing a coordinated approach to strengthening rural distribution and retention of doctors

#### Fragmented governance structure and the absence of an HRH unit

Respondents noted extensive fragmentation in governance structures within the state, and nationally, that made coordinated approaches to rural retention and distribution, and HRH policymaking more broadly, challenging.

*“Uttar Pradesh is the only State where in the Medical Health department there is medical health, family welfare, medical education, Ayurved unani are made into 4 parts and all 4 have ministers, there are 4 principal secretaries (Pramukh Sachiv) … There is nothing like this anywhere else in India.” FGD2, Health worker association.*

#### Addressing perceived corruption and weak accountability in the health sector

Respondents spoke openly about their perceptions of the lack of strong accountability mechanisms and the existence of corruption in postings, transfers and other HR processes such as promotion, benefits, leave, etc. Such corruption in their view lead to major difficulties in both attracting candidates and also ensuring that candidates are deployed and retained in rural areas. Fully addressing the types of corruption noted by respondents would involve accountability or other policy interventions that would likely face considerable opposition from those cadres negatively impacted by such measures. For example, respondents noted the need for regular transfers of clerical officers at frontline facilities, district and sub-district offices in order to curb corruption within HR processes.

In response to a question about the impact of perceived corruption on HRH policy, a district administrator noted,

*“If these kinds of things [corruption] happen then the interest to work also starts to diminish...In my mind I am thinking that my children are living far away from me and I should be able to meet them at least once in a week. But I am unable to get any leave for weeks. So there are lots of problems …” FGD3 District-level administrators.*

*Power dynamics and health worker associations:* The power of health worker associations to facilitate or impede certain policies was as an important factor in developing and implementing a comprehensive strategy to rural retention and distribution. Doctors’ associations were reported to be particularly powerful in influencing the process.

*“If [government doctors’ association] opposes this then it cannot go further. The government doesn’t have so much power to go against [the government doctors’ association] and implement a policy.”* IDI1 (Health systems expert).

A few respondents noted that other health worker associations also wielded power – albeit to varying degrees – and could significantly impact policy processes. For example, one respondent discussed how the association representing clerical officers could stymie the initiative to regularly transfer the cadre in order to reduce corruption within HR processes.

#### *Phasing and timing of policies within the bundle*

Some respondents noted that certain policies need to be implemented jointly with others in order to meet the goal of rural distribution and retention. For example, a representative of a doctors’ association noted that a policy around rural postings should ideally be complemented by investments in staff housing and good quality options for educating school-aged children.

Conversely, a few respondents noted the potential for adverse outcomes when bundling certain policies together, such as home district posting and permitting dual practice.

*[Respondent explaining opposition to home district posting] “Because one, they will get to be near their relatives and friends, and going home will be on their mind, they will think about going home. Their efficiency will reduce; more focus will be given on private practice …*.” *IDI14 (Rural medical practitioners’ association).*

#### Opportunities for reform

Despite the many challenges, some respondents also noted that there were several opportunities to strengthen HRH policy in the state. Successes in improving rural distribution and retention from other Indian states and other countries with similar health systems provided roadmaps for decision-makers to adapt. The growing focus on primary care through recent policy changes at the national and state level signaled an important opportunity for strengthen availability of services at these facilities, provided that various departments could coordinate with one another effectively.

## Discussion

Ensuring the availability of health workers in rural areas is a persistent challenge in India and other LMICs. Recognizing the complex, interlinked nature of the problem, normative guidance increasingly focuses on ‘bundled’ interventions which utilize a range of policy measures to more effectively address the issue [10, 40, 41]. Adopting this type of coordinated approach to health policy is challenging in any context, and arguably, more so with HRH policy. HRH policy is often complex, due in part to governance bottlenecks, power hierarchies amongst and between health workers and administrative cadres, and ineffective or weak systems of accountability and transparency [42–44]. However, few studies have explored stakeholder perspectives across a range of HRH policies [17], and even fewer specifically explored issues of rural distribution and retention [14].

Our analysis reveals several barriers to developing and implementing holistic policies to strengthen rural distribution and retention in UP, India. We discuss three key findings here -.

The lack of coordinated and effective governance in the health sector is a major barrier to improving rural retention and development in UP, an issue that has emerged in the development of HRH policy in other LMICs, such as Bangladesh [14] and South Africa [17]. Our study builds on this literature by describing the disconnects between various units within the health sector, and between health and other sectors. Rural areas in Uttar Pradesh continue to face considerable inequities in access to quality, affordable health care, education and other social services, as well as limitations in coverage of transportation, utilities and commerce. These challenges have long been identified as major barriers to both supporting students from rural areas to gain admission to medical colleges, and also attracting health workers to live and work in these regions [9]. The multisectoral action needed to improve these conditions was found to be lacking in this context, as it has in other settings and other policy issues [9, 45].

The impact of power asymmetries amongst stakeholders in shaping rural retention policy has been previously discussed [14, 46]. Our findings add to this literature by highlighting the policy role of health worker associations, particularly various doctors’ associations. Those policies that were contested by doctors’ associations (i.e., AYUSH task shifting) would require considerable political capital to be adopted and implemented. Given the mix of policies required for improving rural retention and distribution of doctors, decision makers would have to carefully plan the selection and timing for formulating and adopting policies, particularly those that could result in opposition or blocking from health worker associations.

Finally, stakeholders discussed major challenges in implementation due to inefficient mechanisms for accountability and perceptions of widespread corruption across the health system. Similar issues have been identified in HRH policy in other regions of India [47], as well as other LMICs [42, 48–50]. Addressing these accountability concerns may require additional policies – for example, regularly transferring clerical cadres responding for HR processes – that would likely result in resistance from their representative associations. In other instances, the development of complex policies such as the regulation of private practice would require careful end-to-end planning, particularly in terms of enforcement and accountability, in order to avoid the emergence of new problems brought about directly as a result of the policy. The growing literature documenting anti-corruption, accountability and transparency policies in LMIC health sectors would offer important insights for stakeholders working to develop and implement HRH policy [51–53].

Nevertheless, UP is at a unique moment in the development of HRH policy. At the national and state level, there appears to be strong commitment to expanding rural distribution and retention of doctors, and considerable resources invested in expanding supply of health workers, including doctors. Several important policy decisions have already been taken with regards to the policy challenges noted in this paper. For example, the state has transitioned from the system of having Chief Medical Officers post medical officers, to the state managing these postings centrally. Similarly, the state has taken steps to improve the quality of nursing in the public sector by increasing the level of qualifications for new recruits. These developments suggest a window of opportunity in the near term to develop and implement policy solutions.

### Policy implications

Our analysis has several implications for policy. We modified the initial policy proposals and also identified new approaches that addressed concerns raised during the analysis (see Table 5). At the time of writing, plans for further consultative processes to formalize these policy options have been disrupted by the COVID-19 pandemic; we hope to re-initiate them as circumstances allow. Here, we present approaches for addressing the issues laid out in the analysis.

**Table 5:** Additional/revised policies and approaches based on stakeholder analysis findings

1. Reduction of compulsory service period for newly hired doctors from three to two years	
2. Development of ‘cluster’ housing for staff members in larger towns within districts (from which staff can travel to their workplaces)	
3. Work with the Education Department to develop and implement policies for the education of public sector health staff’s children at central government schools	
4. Organize consultative process for a comprehensive range of stakeholders to provide inputs on the HRH strategy	

It is apparent that opposition to specific policies (in the case of UP these included compulsory rural postings and AYUSH task shifting) implies the need for careful negotiation and/or high-level political support in order to pass these policies in the face of opposition. Policymakers should also consider bundling interventions strategically, pairing a policy that will likely meet with resistance, with one that will be favorably received (home district posting and private practice, for example, in the UP case).

While GoUP may have particularly acute challenges in terms of fragmented governance for health this is a common problem across other states and countries [45]. For HRH issues in particular, it will be important to examine existing health and multisectoral governance structures and identify areas for improvement. For example, a unit focused specifically on HRH policy that coordinates across various ministries, departments and agencies would be a first step in identifying and addressing gaps and bottlenecks in the policy process. Such a unit may also be tasked with looking within and beyond the health sector for particular policy levers that can address various challenges with rural deployment and distribution.

Finally, it is widely understood that policy adoption does not translate to effective policy implementation, and as part of any package of HRH policies it will be necessary to rigorously plan for, monitor and evaluate policy rollout and build in accountability measures and safeguards. These include regular feedback mechanisms for improving policies, processes for oversight (including by community members) and transparent information systems [54].

Our study had several limitations – 1) While we identified an extensive list of stakeholders, we were unable to secure interviews with certain key stakeholders due to scheduling conflicts and logistical issues, particularly in the case of senior government officials. As a result, these findings should be interpreted recognizing this limitation in our sampling; 2) Due to the narrow timeframe in which to conduct the study, we could not conduct extensive interviews and focus groups in rural locations within UP; 3) Given the extensive nature of the HRH strategy, respondents could not address all policies in the context of the interviews; 4) Respondents discussed their perceptions of corruption in the system, but we did not independently conduct research to verify their claims; 5) We had intended to conduct a member checking exercise as part of a consultative process with stakeholders during the finalization of the HRH strategy, but have delayed this step due to the COVID-19 pandemic.

## Conclusion

In this paper, we examined stakeholder perspectives on the development of policy to strengthen the distribution and retention of doctors in rural UP, India. We have three main conclusions from this analysis – 1) adopting a coordinated approach to rural retention and distribution policy is negatively impacted by institutional fragmentation, the absence of dedicated HRH policymaking units, and other health and social sector governance challenges in UP; 2) the opposition to these policies by health worker associations creates difficult conditions for decisive, wide-ranging policy development; 3) regardless of policy adoption, policy implementation is severely hampered by weak mechanisms of accountability and pervasive corruption at local, district and state level, necessitating fresh thinking around effective, transparent implementation strategies.

## Supplementary Information

**Additional file 1.**

## Data Availability

The datasets generated and/or analyzed during the current study are not publicly available due to the sensitive information contained within many interviews, but are available from the corresponding author on reasonable request.

## Abbreviations

AYUSH: Ayurveda, Yoga & Naturopathy, Unani, Siddha and Homoeopathy
GoUP: Government of Uttar Pradesh
HRH: Human Resources for Health
LMICs: Low- and middle-income countries
UP: Uttar Pradesh

## Footnotes

1 Key informants included officials from the UP Department of Medical Health and Family Welfare, frontline health workers (medical officers and staff nurses) in the department as well as partner development agencies that work closely with the government for health system strengthening in UP. These discussions were conducted to develop the initial policy bundle prior to the stakeholder analysis.

2 The Department of Medical Health and Family Welfare in Uttar Pradesh was merged from two separate departments – Department of Medical Health and Department of Family Welfare – in 2020, following the time of data collection. We have used its current name in the paper.
