# Supplementary material for: Stakeholder perspectives on proposed policies to improve distribution and retention of doctors in rural areas of Uttar Pradesh, India
Source: BMC Health Serv Res. 2021 Sep 29;21:1027. doi: 10.1186/s12913-021-06765-x (PMC8478638; doi:10.1186/s12913-021-06765-x)
Supplement: Supplementary file 1 — Additional file 1. [file 12913_2021_6765_MOESM1_ESM.docx]

**“Doctors will love….but that is not in the interest of the public”: stakeholder perspectives on proposed policies to improve distribution and retention of doctors in rural areas of Uttar Pradesh, India**

Veena Sriram (Corresponding Author)

University of British Columbia

School of Public Policy and Global Affairs and School of Population and Public Health

C. K. Choi Building

251 – 1855 West Mall

Vancouver, B.C., V6T 1Z2, Canada

Email: [veenasriram1@gmail.com](mailto:veenasriram1@gmail.com)

Shreya Hariyani

Johns Hopkins Bloomberg School of Public Health

Ratan Square, Vidhan Sabha Marg, Lucknow

Uttar Pradesh, India

Email: shreya.hariyani@jhu.edu

Ummekulsoom Lalani

Johns Hopkins Bloomberg School of Public Health

615 N. Wolfe Street

Baltimore, MD, USA

Email: umme.lalani@gmail.com

Ravi Teja Buddhiraju

Uttar Pradesh Technical Support Unit,

India Health Action Trust, Ratan Square, Vidhan Sabha Marg,

Lucknow, Uttar Pradesh, India

Email: ravi.teja@ihat.in

Pooja Pandey

Indian Administrative Service

Lucknow, Uttar Pradesh, India

Email: [poojapandeylko@gmail.com](mailto:poojapandeylko@gmail.com)

Sara Bennett

Johns Hopkins Bloomberg School of Public Health

615 N. Wolfe Street

Baltimore, MD, USA

Email: sbennett@jhu.edu

**Appendix 2: Focus Group Discussion Guide^[[1]](#footnote-1)^**

Date: ___/___/_____

ID #: _____

Group Facilitators: ________

Note taker:

Start time:

End time:

**Facilitator Note:** Facilitators are asked to complete as many of the below questions as possible given the allotted time for the FGD. Facilitators may ask additional or follow-up questions in the event that information on a particular topic that has not been elicited from the below questions.

**Introduction:**

We are from Johns Hopkins Bloomberg School of Public Health and we are working with the Department of Medical Health and Family Welfare in Uttar Pradesh to develop a comprehensive human resource policy for the state. As part of that effort, we are conducting a study to understand the opinions of important stakeholders in the state regarding this proposed policy. As important stakeholders in this sector, it is crucial for us to obtain your opinion. We plan to conduct about 20 interviews and focus groups discussions to produce a general report on the opinions of the major health sector actors. The information obtained through these interviews and discussions will be for the use of the policy research team without identifying individual opinions. We also encourage you to express your thoughts and opinions freely. In addition, we would like each of you to respect each other’s privacy and keep the conversations we have today confidential.

**<Consent Processes>**

**Background characteristics of FGD Participants**

<Each participant will be asked to state their current role, location and length of time in position>

**Discussion Guide**

What are the key health workforce challenges in the state?

If you had to propose one major policy change to address health workforce issues in the state, what would it be?

1. Earlier, we had talked about a proposed HRH policy for the state. As part of the proposed policy we are considering a number of potential policy changes. [Share document or project PPT] We would like to talk through these policy options. [Ask the following questions for each relevant option]

What are your perspectives on this suggested policy change?

(Probe: Advantages/ disadvantages)

Would you be supportive of this policy change? Please explain your answer. *[Probe regarding any differences between individual and organizational views]*

If you are a member of an association, what would the views of your association be on this policy?

How would they demonstrate their support/opposition?

Are there any groups that the association would work with to express its support/opposition?

How can we ensure that the adoption and implementation of this policy successful?

Are there particular stakeholders whose support is important for this policy to succeed? If so, please list these stakeholders.

What challenges do you anticipate in adopting or implementing this policy?

Are there any stakeholders who would oppose this policy? Please explain your answer.

What other policy options should we consider to address the underlying problem?

Do you have any other ideas regarding the proposed HRH policy for the state?
Based on our discussion, are there any other individuals or organizations who are important stakeholders for these policies that you would recommend us speaking with?

Thank you for your time.

1. I Focus Group Discussion guides are also available in Hindi and can be shared upon request [↑](#footnote-ref-1)
